# Nanoemulsion is an effective antimicrobial for methicillin-resistant *Staphylococcus aureus* in infected swine skin burn wounds

**DOI:** 10.1128/spectrum.01378-24

**Published:** 2024-10-14

**Authors:** Zhengyi Cao, Jesse Chen, Jayme Cannon, Zachary Meyer, Yongqing Li, Wenlu Ouyang, James Baker, Su He Wang

**Affiliations:** 1 Michigan Nanotechnology Institute for Medicine and Biological Sciences, University of Michigan, Ann Arbor, Michigan, USA; 2 Department of Surgery, University of Michigan, Ann Arbor, Michigan, USA; 3 Department of Internal Medicine, Division of Allergy, University of Michigan, Ann Arbor, Michigan, USA; Seton Hall University, South Orange, New Jersey, USA

**Keywords:** thermal injury, porcine infection model, nanomaterials, antimicrobial agents

## Abstract

Burns are one of the most common injuries in both civilian and combat settings and are difficult to treat. This is particularly true when the wounds are infected with antibiotic-resistant bacteria such as methicillin-resistant *Staphylococcus aureus* (MRSA). A new generation of safe, broadly effective, and easily applied anti-infection agents is needed to successfully prevent and treat infections. Nanoemulsions (NEs) are nanometer-sized particles with a positively charged surfactant at their oil-water interface. In the current study, we further investigated antimicrobial NEs as a treatment to address burn wounds infected by MRSA. Specifically, using a porcine skin model, we infected partial thickness thermal burn wounds with MRSA and then treated it with the nanoemulsion formulation (NB-201) or placebo controls. Bacterial viability after treatment was determined, and inflammation indexes in wounds were scored by histopathology. Topical treatment of infected wounds with NB-201 resulted in reduced colony-forming units (CFUs) compared to placebo treatment. In addition, NB-201 was effective in significantly alleviating inflammation in the treated wounds and promoting wound healing. These results indicate that NB-201 is a promising new agent to treat skin burn wounds infected with MRSA.

**IMPORTANCE:**

The findings of this study are focused on therapeutic applications of nanotechnology. In the current study, we demonstrated that a nanoemulsion formulation could effectively kill methicillin-resistant *Staphylococcus aureus* (MRSA) infection in porcine skin burn wounds. Infection of MRSA in burn wound is a common threat to public health and is usually difficult to treat due to limited therapies available. NB-201 was effective in significantly alleviating inflammation in the treated wounds and promoting wound healing. Therefore, the finding of this study has a great potential to make this formulation a novel antimicrobial agent against MRSA.

## INTRODUCTION

Over 300,000 burn injuries occur every year in the United States and need medical attention (1). Severe burns can lead to disability or even mortality especially due to subsequent complications and infection. Of particular concern are burn wounds infected with antibiotic-resistant organisms. Developing new topical agents for preventing and treating burns complicated by resistant bacteria remains an important goal.

*Staphylococcus aureus*, a Gram-positive bacterium, is widely present in the skin of both humans and animals. Importantly, the number of individuals colonized or infected with methicillin-resistant *Staphylococcus aureus* (MRSA) is increasing. It has been reported that almost 30% of the human population carries MRSA, which is a potential cause of severe wound infections associated with high morbidity and mortality (2, 3). This extends to a range of complex MRSA infections, including skin and soft tissues, pneumonia, bone and joint infections, osteomyelitis, and infective endocarditis (4).

MRSA is a problem in a high percentage of infected skin burn wounds (5, 6). This is likely due to burn patients losing their skin barrier, having decreased numbers of bactericidal polymorphonuclear cells, and being relatively immunodeficient (6, 7). Without proper treatments, MRSA can rapidly disseminate from burn wound sites into the blood stream. Therefore, burn patients with MRSA infection have significantly increased risk of bacteremia, septicemia, and serious clinical complications including the loss of skin grafts (5, 6). The outcome for these patients is often fatal. Currently, there are a few drugs, such as vancomycin, that effectively treat MRSA infection. However, MRSA resistance has been recognized to develop with all current treatments (8, 9), limiting therapeutic options. Furthermore, several MRSA antibiotics can cause significant side effects such as renal toxicity and hepatotoxicity (8, 9). Therefore, there is a compelling need to develop novel agents and methods to overcome the treatment resistance and combat MRSA infections in burn patients.

An antimicrobial nanoemulsion formulation, NB-201, was previously shown to be an effective topical antimicrobial for *Staphylococcus aureus* in a murine abrasion wound model (10). The effectiveness of NB-201 against multidrug-resistant bacteria was also subsequently demonstrated in treating wounds infected by MRSA, vancomycin-resistant *Enterococcus* (VRE), and polymicrobial infections (MRSA + VRE) in a porcine abrasion wound model (11, 12). However, this approach has not yet been tested in skin burn wounds infected with MRSA in the pig model, which more closely approximates human skin wounds. Also, skin wounds infected with MRSA have increased inflammation with elevated pro-inflammatory cytokine concentrations (11). In addition to skin tissue cytokines, circulating citrullinated histone H3 (CitH3) can be utilized as another biomarker of wound inflammation and sepsis caused by infection (13, 14). This involves peptidyl arginine deiminase 4 (PAD4), which initiates neutrophil extracellular trap (NET) formation, or NETosis (14, 15). The level of CitH3 is therefore correlated with the degree of infections of Gram-positive bacteria (15, 16). Together, these assays enable us to analyze anti-inflammatory efficacy to better control MRSA infection.

In this study, NB-201 was tested as a topical antimicrobial to treat burn wounds infected with MRSA in a porcine skin burn model. This model involves a partial thickness thermal burn that results in highly reproducible damage to the upper 30% to 50% of the dermis (17). Our data showed that NB-201 was able to reduce MRSA colony-forming units (CFUs) in infected burn wounds, inhibit dermal inflammation, and promote wound healing. NB-201 is a promising agent that can be utilized to combat MRSA in skin burn wounds and overcome resistance.

## MATERIALS AND METHODS

### Formulation of NB-201

Nanoemulsions (NEs) are oil-in-water emulsions formulated from soybean oil, water, solvent, cationic surfactants, and benzalkonium chloride (BZK). NB-201 was manufactured by emulsification under high-energy homogenization using high shear conditions of super refined soybean oil, water, glycerol, EDTA, Tween 20, and 0.2% BZK as previously described (10, 11). The resultant formulation had a mean particle diameter of 350 nm with the positive surface charge of X as determined by Zeta potential. A placebo (X-1739) for NB-201 was manufactured in the same manner but lacked BZK.

### Bacterial strain

MRSA strains SA31 (clinical isolate; University of Michigan) and BAA-1680 (American Type Culture Collection) were used to establish a contaminated wound. This was done to evaluate the effectiveness against both a reference strain and a true skin pathogen.

### Creation of a partial thickness thermal burn

**Fig 1:**
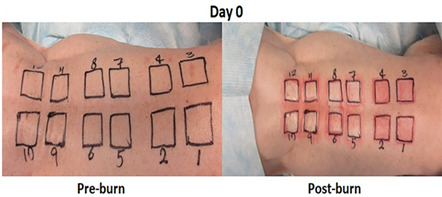
Pig partial thickness thermal burn infected with MRSA. The treatment schedule allowed for application of NB-201 or the placebo three times per week for 2 weeks.

A partial thickness thermal burn was used to create a highly reproducible wound to the upper 30% to 50% of the dermis (17). The process of the burn wound generation is as described below. Under general anesthesia in the prone position, we removed flank and back hair from the pig using Nair Hair Remover Lotion. The skin was prepared with 1–2% chlorhexidine gluconate and 70% isopropyl alcohol solution followed by draping. A partial thickness thermal burn was created on the pig paravertebral area by applying a 3 × 3 cm, brass bar preheated in hot water to 80°C. The bar is applied for 20 seconds, in the vertical position, with contact pressure being supplied by gravity. In a typical study, twelve 3 × 3 cm wounds were created on eight pigs' paravertebral skin area (Fig. 1).

### Infection of thermal burn wound and treatment with nanoemulsions

Each burn wound was inoculated with 3–5 × 10^7^ CFUs of MRSA (SA31 or BAA-1680) re-suspended in phosphate-buffered saline. Subsequently, the treatment protocol was scheduled as follows: Day 0 procedures included wound creation, MRSA infection, and application of nanoemulsion; on Days 1, 2, 5, 7, 9, and 12, the wounds were swabbed each day for colony counts followed by application of treatment; on Day 14, the animals were euthanatized, and wounds were swabbed a final time and then punch biopsied.

The specific treatment was as follows. Two hours after the inoculation, four of the twelve wounds were sprayed with 20% NB-201, four wounds were sprayed with NB-201 placebo, and four were left untreated. Each 3 × 3 cm treatment area was swabbed and then swabs were each placed in 50 mL tubes with 500 µL of LB media. Swabs were incubated for 1 hour, on a rocker, at 4°C. Swabs were then discarded, and the remaining LB media was plated. At each time point and treatment condition, the dilution factors of the samples plated varied based on group. For some studies, we also compared NB-201 with silver sulfadiazine (SSD) treatment in the same setting, a typical topical antimicrobial agent used clinically for burn and injury wounds (18, 19).

### Evaluation of colony-forming units

The swabbed samples were evaluated for quantitative bacterial culture under two conditions: an MRSA screen plate containing oxacillin (6 µg/mL) and a non-selective plate without antibiotics. Plates were incubated for 24 hours at 37°C, and CFUs were counted in samples obtained from the skin burn wounds treated NB-201 or placebo at four time points (Days 1, 2, 7, and 14) to monitor the effect of NB-201 on MRSA CFU.

### Evaluation of tissue inflammation

Inflammation indexes were assessed by histopathology. Porcine punch biopsy specimens from each treatment group were examined to assess the degree of necrosis, inflammation, and healing using a 0–6 scoring system adapted from previous wound healing studies (11, 12). All sections were fixed in formalin and processed for histological examination. Evaluating and scoring for necrosis and inflammation were categorized to the epidermal, dermal, and deep levels, as well as epidermal hyperplasia (Table 1).

**TABLE 1:** Grading scheme for evaluation of pig skin sections

Epidermal necrosis	Dermal necrosis (µm)	Superficial dermal inflammation
0 = none	0 = none	0 = none
1 = separation	1 ≤ 100	1 = mild
2 = complete	2 = 100–300	2 = moderate
3 = missing	3 = 300–500	3 = severe
	4 ≥ 500	4 = necrotizing
		5 = locally extensive necrotizing
		6 = diffuse necrotizing

Dermal inflammation granulation	Deep inflammation	Epidermal hyperplasia
0 = none	0 = none	0 = none
1 = mild multifocal	1 = mild	1 = mild
2 = moderate multifocal	2 = moderate multifocal	2 = moderate
3 = mild diffuse	3 = moderate locally extensive	3 = severe
4 = moderate diffuse	4 = severe	4 = extreme
5 = severe		

### Determination of pro-inflammatory cytokines

Both the swabbed samples and punch biopsies were used to evaluate the level of inflammation. Punch biopsies were homogenized and suspended in 0.01% Triton and proteinase inhibitor buffer. The concentrations of three pro-inflammatory cytokines, IL-1β, IL-6, and IL-8, were measured using DuoSet ELISA kits according to the instruction of the manufacturer (R&D Systems, MN).

### Measurement of CitH3 level

To quantify CitH3 in the skin tissue lysate, we employed an ELISA. First, 0.5 µg/well CitH3 monoclonal antibody was coated in 96-well plates at 4°C overnight, and the wells were subsequently blocked with 100 µL of protein-free blocking buffer at 4°C overnight. To produce the standard curve and measure the concentration in the lysate, we incubated the wells with synthesized CitH3 peptide and skin tissue lysates, respectively, for 2 hours at room temperature (RT). Next, rabbit anti-CitH3 polyclonal antibody was added and incubated for 2 hours at RT. The plates were then probed with anti-rabbit IgG, conjugated to horseradish peroxidase. Finally, the plate was developed for 30 minutes at RT with 3,3′,5,5′-tetramethylbenzidine. Stop solution was added, and absorbance at 450 nm was recorded.

### Statistical analysis

Data were analyzed using a two-tailed unpaired *t*-test with Welch's correction and one-way ANOVA, and *p*-values of less than 0.05 were considered statistically significant.

## RESULTS

### Evaluation of NB-201 formulations against MRSA-infected burn wound

**Fig 2:**
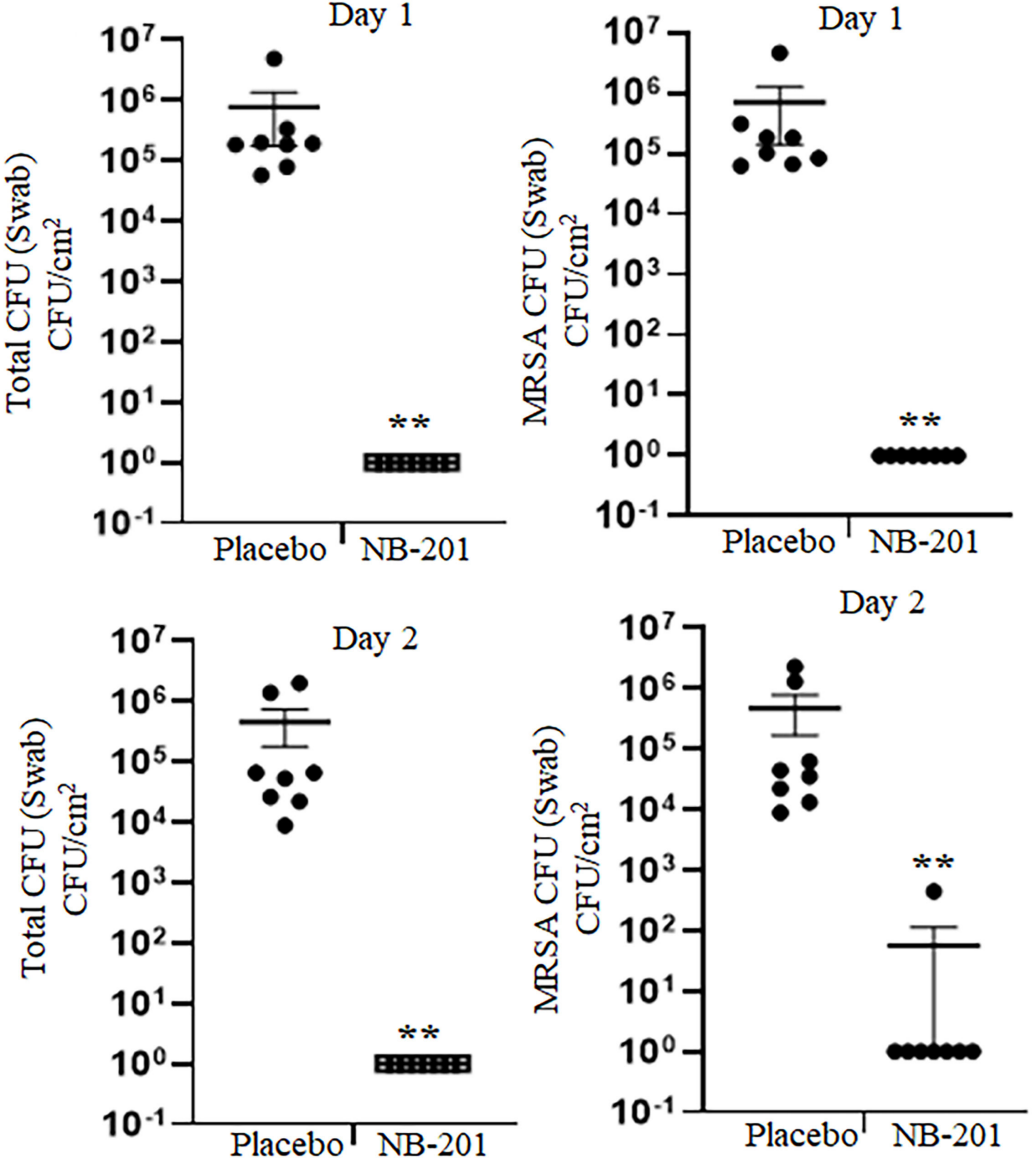
Inhibition of MRSA in the porcine skin burn model by NB-201 (Days 1 and 2). A partial thickness thermal burn was created on the porcine paravertebral area by applying a brass bar preheated to 80°C. Two hours after the MRSA inoculation, the burn wounds were sprayed with 20% NB-201 or NB-201 placebo. The treatment area was swabbed and then swabs were tested for colony growth. While all wounds sprayed with NB-201 had no colony growth, the colonies grew in the wounds treated with the placebo at both Days 1 and 2 (*n* = 8; **P < 0.01).

**Fig 3:**
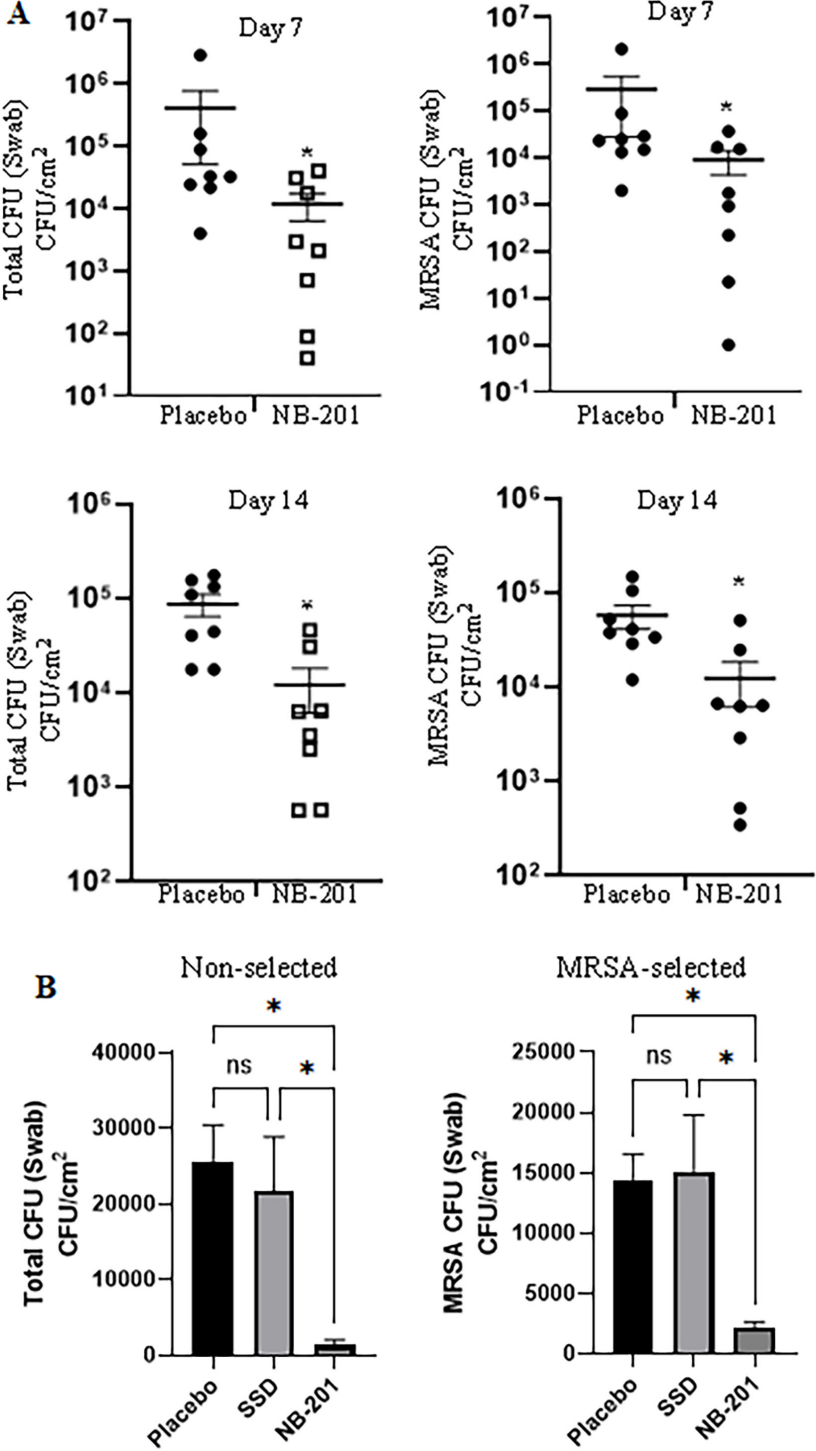
Inhibition of MRSA in the porcine skin burn model by NB-201 (Days 7 and 14). A partial thickness thermal burn was created on the porcine paravertebral area by applying a brass bar preheated to 80°C. Two hours after the MRSA inoculation, the burn wounds were sprayed with 20% NB-201 or NB-201 placebo. The treatment area was swabbed and then swabs were tested for colony growth. (**A).** All wounds sprayed with the placebo showed significantly more colony growth compared with NB-201 at both Days 7 and 11 post-treatments (*n* = 8, *P < 0.05). (**B).** Compared with SSD or placebo, NB-201 treatment significantly reduced the colony growth on both MRSA-selected and non-selected plates (*n* = 4; *P < 0.05) while there was no difference between SSD and placebo.

Two days after the wound was infected with MRSA, we examined it for evidence of infection. Total skin bacterial load was determined by nutrient agar plates, and MRSA screen plates that included oxacillin were used for MRSA colony selection. At 1-day post-burn injury with MRSA infection, all wounds treated with nanoemulsion NB-201 had no colony growth on either the non-selective plate or the MRSA screen plate, whereas all wounds sprayed with the placebo showed colony growth on the non-selective and MRSA screen plates (*n* = 8; **P < 0.01) (Fig. 2). At Day 14 post-burn injury with MRSA infection, all wounds treated with NB-201 had significantly less colony growth on both the non-selective plate and the MRSA screen plate, compared with those sprayed with the placebo (*n* = 8; *P < 0.05) (Fig. 3A). Our data showed that the topical treatment with NB-201 could significantly decrease skin tissue bacterial loads. In addition, we found that SSD had a minimal effect on the colony growth as its effects were not significantly different from the placebo control (Fig. 3B).

### Analysis of pathology alternations by NB-201 in MRSA-infected burn wound

**Fig 4:**
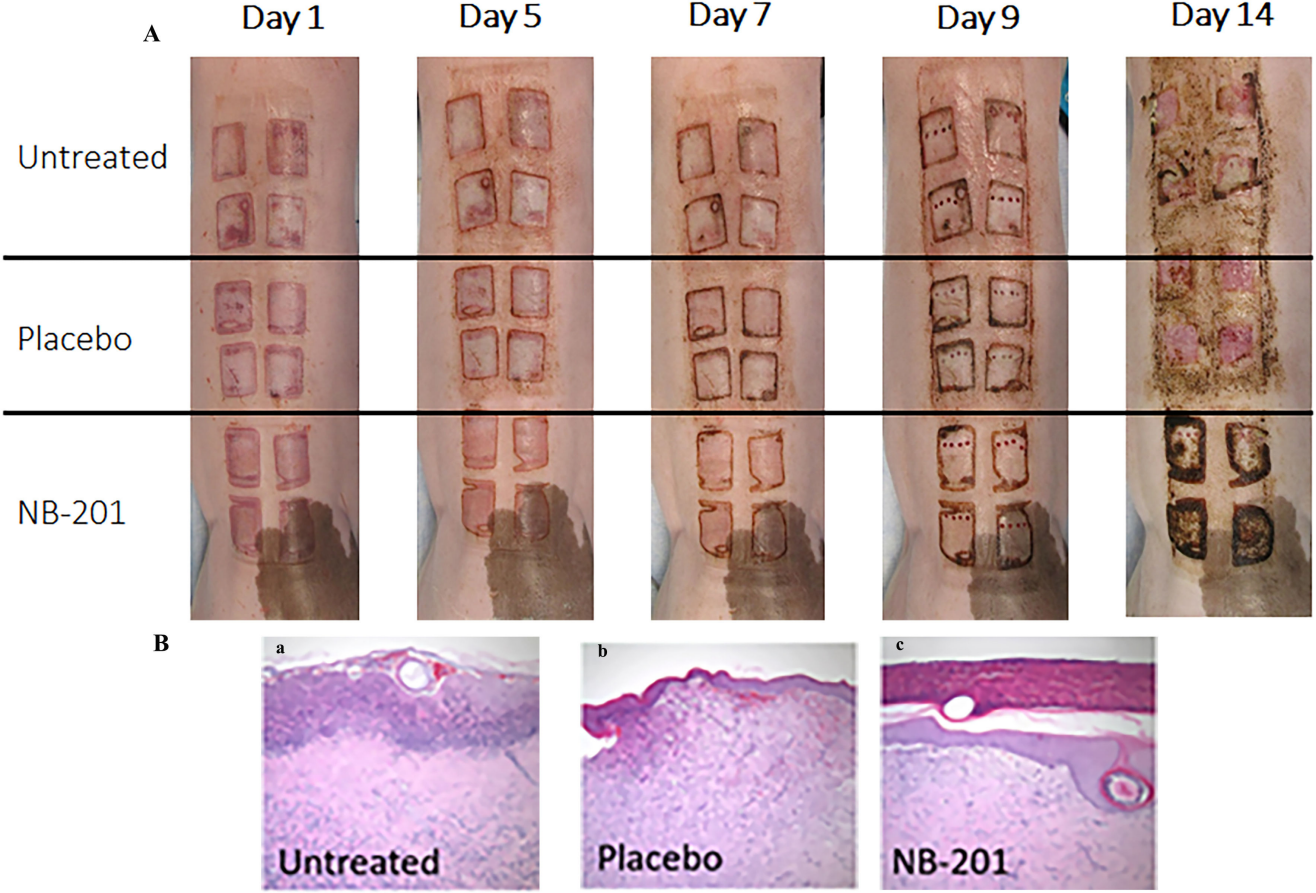
Photographs of infected burn wounds and their histologic staining. (**A)**. Digital photographs of infected burn wounds in the porcine skin burn model. Less inflammation and faster healings were observed in NB-201-treated wounds compared with placebo and untreated wounds. (**B)**. Representative hematoxylin and eosin (HE) stained histologic section of infected burn wounds. (**A).** Untreated. (**B).** Placebo. (**C).** NB-201. NB-201-treated wound at Day 14 showed faster healing than placebo and phosphate-buffered saline. The black tone showed is not necrotic hallmark, but healed crust demonstrated in HE stained histologic section.

Gross pathological changes of the burn wounds were recorded by digital photograph and analyzed by a blinded pathologist. There was less inflammation and faster healing observed in NB-201-treated wounds compared with placebo or saline (Fig. 4). At Day 14, scars were obviously formed in NB-201-treated wounds, indicating that wound healing entered the final remodeling phase. However, no scars were observed in two control wounds and the wounds showed redness, some edema, and pus (Fig. 4).

**Fig 5:**
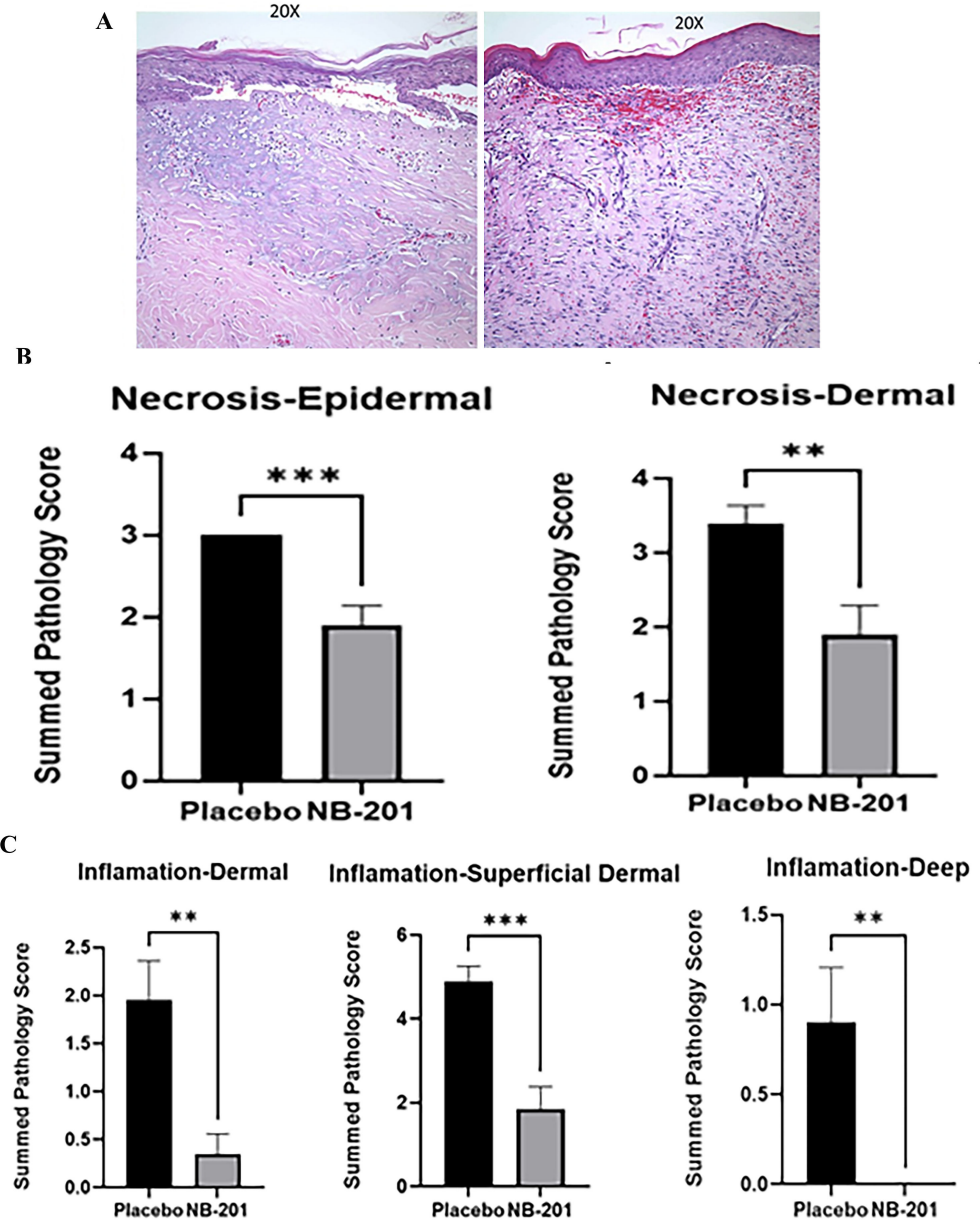
Histologic imaging of MRSA-infected wounds and their pathological analysis. (**A)**. Histologic imaging of MRSA-infected wounds treated with NB-201 or placebo. Epidermal and dermal necrosis (left panel) and inflammation (right panel). (**B)**. Both epidermal and dermal necrosis are significantly reduced in the NB-201-treated wounds compared to placebo (*n* = 8; **P < 0.01).** (C)**. All three categories of inflammation are significantly reduced in the NB-201-treated wounds compared to placebo (*n* = 8; ***P < 0.001).

Punch biopsy skin specimens obtained at Day 14 showed NB-201 treatment attenuated the neutrophil sequestration and clearly inhibited both epidermal and deep dermal inflammation compared with control groups. NB-201 was particularly effective in alleviating deep dermal inflammation, which was absent in most treated wounds (Fig. 5). These pathological observations supported the visual assessment and the formation of scars in NB-201-treated wounds (Fig. 4).

**Fig 6:**
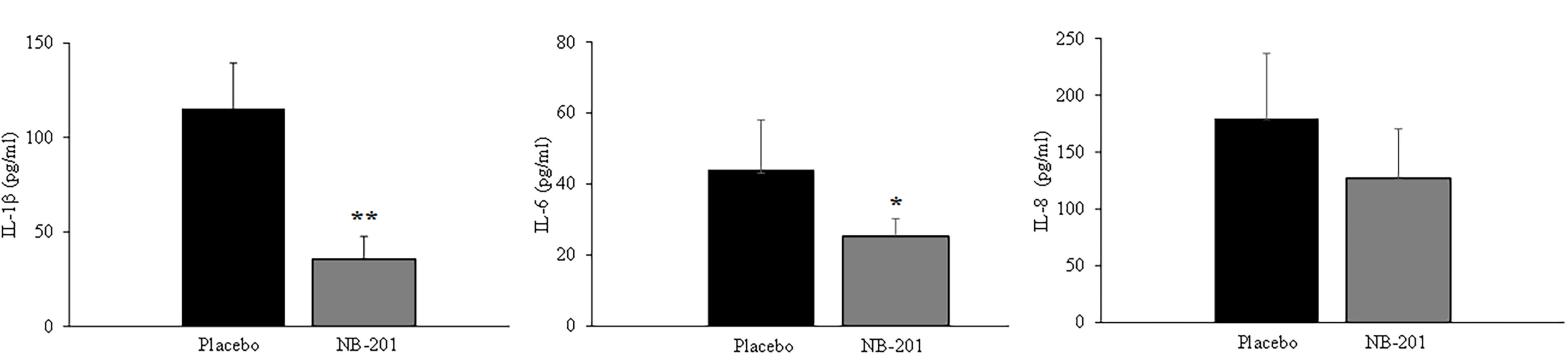
The inflammation cytokines IL-1β and IL-6 in the infected burn wounds of the porcine skin burn model. The levels of IL-1β and IL-6 cytokines in wound tissue samples were reduced significantly at Day 7 compared with the placebo (*n* = 8; **P < 0.01, *P < 0.05, respectively). Similar data were obtained in Day 14.

**Fig 7:**
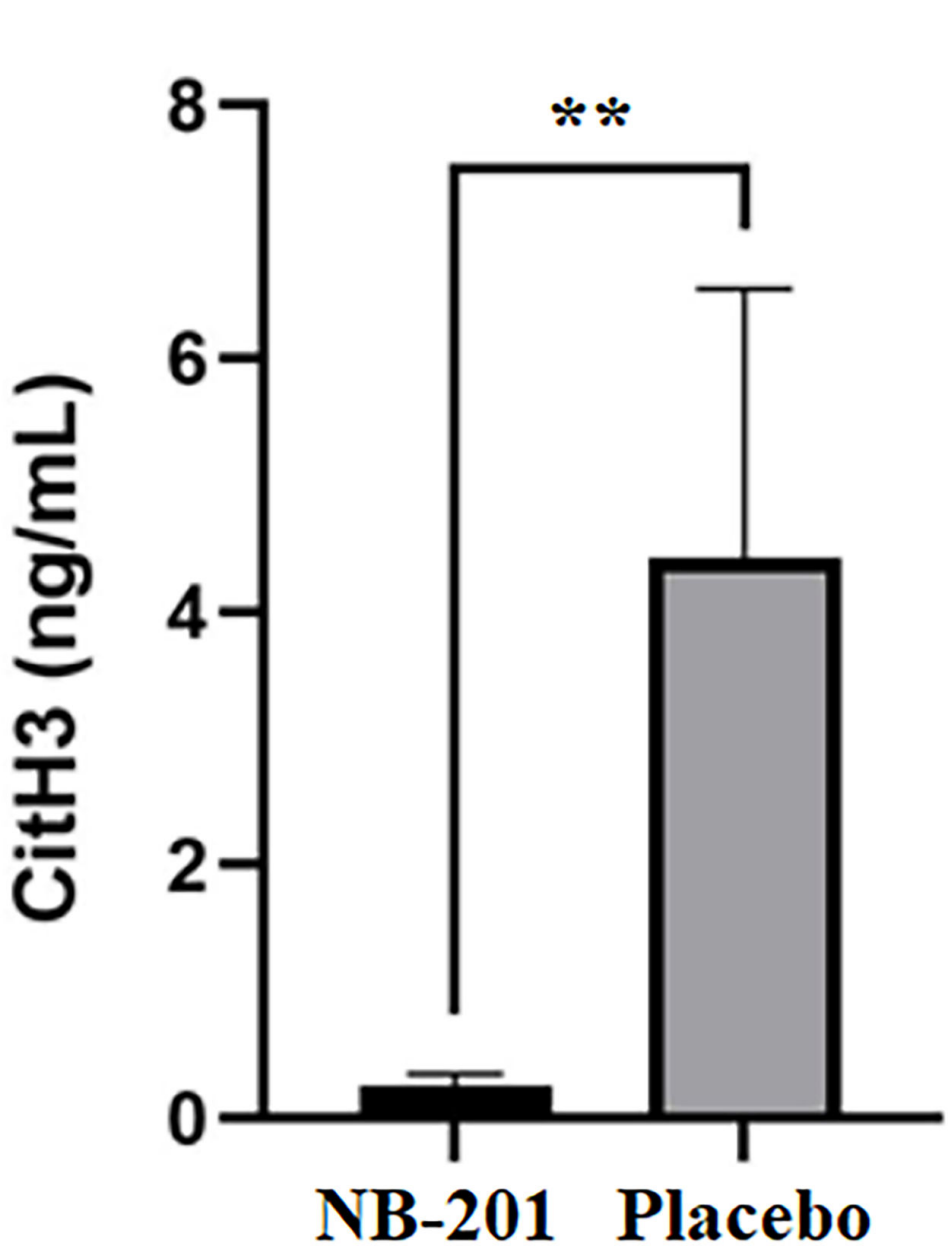
Level of CitH3 from treated wounds. NB-201 treatment significantly reduced the level of CitH3 compared to placebo (*n* = 8; **P < 0.01).

The levels of pro-inflammatory cytokines IL-1β and IL-6 (Fig. 6) as well as CitH3 (Fig. 7) in wounded tissues were significantly reduced in NB-201-treated groups compared with placebo (*n* = 8; **P < 0.01, *P < 0.05, and **P < 0.01, respectively). However, there was not a significant difference for the level of IL-8 between NB-201 and the placebo. Once again, the data of pro-inflammatory cytokines and CitH3 were in line with visual and pathological assessments of the wounds (Fig. 4 and 5).

## DISCUSSION

Burn wounds are a common and devastating form of trauma (20–22). They are also infection prone as the openness of wound, reduced body surface temperature, and exudation of plasma components create a favorable environment for bacterial colonization and proliferation (21, 23). Among various bacteria, *S. aureus* is the most frequent bacterium found in burn wounds and can lead to devastating local and systemic infections (3, 5, 6, 24, 25). The treatment of *S. aureus* burn wound infections has been challenging due to widespread bacterial resistance to antibiotics. Burn patients with MRSA infection are associated with a particularly poor prognosis. Sepsis can develop in a significant number of burn patients with MRSA infection, which can lead to death through multiple organ system failure (26).

Conventional topical agents for anti-infection are not effective for MRSA as they suffer from several drawbacks including poor permeability across the stratum corneum, repeated applications, local irritation at the application site, and systemic toxicities. For example, silver sulfadiazine (SSD), a common topical antimicrobial agents used clinically for burn and injury wounds, has been shown to have minimal or even no effects on MRSA, as it has poor penetration and weak adhesive abilities, resulting in failure to come into contact with microbes (18, 19). The poor efficacy of SSD against MRSA has also been confirmed in this study (Fig. 3B). Although a number of new topical treatments for MRSA wound infections have been proposed, including the application of polycationic chitosan, sulfur (SVI) hybrids, plant extracts containing curcumin, therapeutic microorganisms and topical antibiotics, none of them have entered clinical use due to limited effectiveness and/or lack of direct clinical trials conducted in humans (27–30). Among these new developments, chitosan is one of the attractive agents due to its exceptional physicochemical and biological properties such as less toxicity, biodegradability, biocompatibility, hemostatic, and mucoadhesive properties (31, 32). However, there are challenges restricting the applicability of chitosan, such as issues of purity, solubility, pH, stability, and mechanical properties. In order to overcome these drawbacks, current studies are trying to modify it to form various new derivatives that are yet to be tested and/or verified *in vitro* and *in vivo* (animals and human) (31, 32).

Although the systemically administered antibiotics, predominantly vancomycin, is the standard for treatment of burn wound infections (3, 5, 6, 25), this line of treatment is now tempered by concerns about the development of drug resistance. This has been reinforced by the frequently isolated of vancomycin-intermediate *S. aureus* from wounds in recent years (5, 8, 9). Furthermore, the systemic administration of antibiotics often does not yield the required concentration for effective antimicrobial effect at the local infection site (24, 33). As a result, the current recommendations of the international clinical practice guidelines (CPGs) for the prevention of burn wound infection are to use topical antimicrobials to prevent infection; however, there is currently no ideal topical antimicrobial agent for all clinical scenarios (34). Therefore, there is an urgent need to develop effective and safe treatments to control and eliminate bacterial colonization from burn wounds to prevent systemic dissemination and to facilitate wound healing.

In the current study, we have demonstrated that NB-201 treatment significantly reduced MRSA colonization in infected burn wounds. This was accompanied by reduction in subsequent growth of MRSA. Importantly, this led to attenuated neutrophil sequestration in the wound with reduction or elimination of both epidermal and deep dermal inflammation. In line with the inhibition of MRSA and inflammation, the levels of some pro-inflammatory cytokines such as IL-1β in the burn wound tissues were noticeably reduced in NB-201-treated groups compared with placebo control. These pathological improvements eventually led to a faster wound healing process in NB-201-treated burn wounds.

The previously defined mechanism of anti-MRSA activity of NB-201 also provides reassurance that resistance to this treatment won't develop in MRSA (10–12). Similar to detergent micelles, NB-201 fuses with the outer membrane of the bacteria resulting in rapid lysis of MRSA. Unlike detergents, however, NB-201 does not disrupt the tissue matrix due to its larger particle size. While resistance to detergents is a problem with bacteria that develop a biofilm, an exopolysaccharide matrix produced around themselves to protect the bacteria, the degree to which this is a problem for MRSA is unclear (25, 35–37). Nanoemulsion NB-201 has been shown to penetrate deeply into biofilms due to its small size (10–12); however, in this study, we have not tested the impact of NB-201 on biofilms formed by MRSA due to the short-term study model used. Nevertheless, this important topic should be addressed in the future.

The most important feature of NB-201 wound treatment is the topical route of delivery. Antibiotics delivered via the systemic application not only have poor penetration into infected wound tissues but also cause more side effects. The topical delivery of NB-201 leads to its high concentrations specifically at the site of infected wound, while leading to less systemic toxicity, and minimal exposure in non-infectious tissues. Finally, NB-201 is very stable at room temperature and in all animal models tested so far, and NB-201 treatments have been well tolerated and caused no adverse effects (10–12).

In conclusion, NB-201 can effectively kill MRSA in swine skin burn wounds, while reducing inflammation and accelerating the healing process. Thus, it is worthwhile to further evaluate this nanoemulsion as a topical microbicidal to treat MRSA-infected burn wounds. Nanoemulsions could potentially be combined with other antimicrobials and anti-inflammatory drugs to minimize burn wound inflammation and facilitate the healing process. This could lead to pursue new avenues of antimicrobial treatment.
